# Gaze behavior in social interactions between beach volleyball players—An exploratory approach

**DOI:** 10.3389/fpsyg.2022.945389

**Published:** 2022-10-11

**Authors:** Andre Nicklas, Lisa-Marie Rückel, Benjamin Noël, Matyas Varga, Jens Kleinert, Martin Boss, Stefanie Klatt

**Affiliations:** ^1^Institute of Exercise Training, Sport Informatics, Department of Cognitive and Team/Racket Sport Research, German Sport University Cologne, Cologne, Germany; ^2^Sport and Exercise Science and Medicine Research Group, University of Brighton, Brighton, United Kingdom; ^3^School of Sport, Exercise, and Health Sciences, Loughborough University, Loughborough, United Kingdom; ^4^Institute of Psychology, Department of Health and Social Psychology, German Sport University Cologne, Cologne, Germany

**Keywords:** gaze behavior, social interaction, team sports, beach volleyball, game performance pressure

## Abstract

Previous research has indicated that social interactions and gaze behavior analyses in a group setting could be essential tools in accomplishing group objectives. However, only a few studies have examined the impact of social interactions on group dynamics in team sports and their influence on team performance. This study aimed to investigate the effects of game performance pressure on the gaze behavior within social interactions between beach volleyball players during game-like situations. Therefore, 18 expert beach volleyball players conducted a high and a low game performance pressure condition while wearing an eye tracking system. The results indicate that higher game performance pressure leads to more and longer fixation on teammates’ faces. A higher need for communication without misunderstandings could explain this adaptation. The longer and more frequent look at the face could improve the receiving of verbal and non-verbal information of the teammate’s face. Further, players showed inter-individual strategies to cope with high game performance pressure regarding their gaze behavior, for example, increasing the number of fixations and the fixation duration on the teammate’s face. Thereby, this study opens a new avenue for research on social interaction and how it is influenced in/through sport.

## Introduction

People coordinate their abilities and skills over time and space in various tasks (e.g., passing a ball or building a house) to achieve a common goal (Fasold et al., 2021). Successful collaborations depend on various factors, such as visual and somatosensory information (Sebanz et al., 2006). Previous studies have shown that individuals can anticipate and integrate their partner’s behavior in their own action planning by sharing mental representations of the expected outcome (Knoblich and Jordan, 2002; Jordan and Knoblich, 2004; Marsh et al., 2006). Clark and Krych (2004) showed that instructing another person on how to build a Lego model while seeing the working area leads to fewer errors and less time to finish. In a team sport context, for example, referees coordinate their gaze to officiate the game (Fasold et al., 2021), or players collaborate to score a goal (Klatt et al., 2021b). On a theoretical level, this is called joint action, which can be regarded as any form of social interaction (Sebanz et al., 2006; Knoblich and Sebanz, 2008).

Social interactions are the interplay of auditory and visual cues and aim to exchange information between at least two people (Argyle and Cook, 1976; Gobel et al., 2015; Wolf et al., 2018). Moreover, not only information but also emotions (e.g., anxiety, happiness) can be transferred implicitly or explicitly within social interactions (Hatfield et al., 2014). Notably, individuals with good interpersonal relationships are often likely to adapt to the collective emotions of the group (Tamminen et al., 2016). In the scientific literature, transferring emotions from one person to another is called emotional contagion (see Hatfield et al., 2014; Boss and Kleinert, 2015; Herrando and Constantinides, 2021; for a review). To exemplify, Moll et al. (2010) showed that post-performance emotions could be transferred to the teammates and influence the overall team performance. Specifically, authors found that celebrating a soccer kick with both arms raised increases the likelihood of the player’s team winning the shootout. Another well-known phenomenon associated with emotional contagion is the collective team collapse, which describes a sudden performance drop of the entire team (Wergin et al., 2018). An important factor within this phenomenon is negative emotional contagion. Experiencing negative emotions is associated with individual underperformance (Barsade and Gibson, 2012; Hill and Shaw, 2013). So, negative emotional contagion can lead to more teammates that underperform. For example, player A might feel insecure or anxious after making a mistake. These negative emotions can be transferred to another teammate (Herrando and Constantinides, 2021 for a review) and affect the teammate’s performance. Thus, the transmission of negative emotions can run through the whole team and lead to a collective team performance drop (Wergin et al., 2018).

In non-continuous sports, such as volleyball or tennis doubles, the nature of the game (e.g., breaks between the rallies) allows for a high frequency of social interactions between the teammates. That is why beach volleyball was chosen in this study. In addition, beach volleyball was chosen because there is no coach on the sidelines, which means that the players have to rely on each other for support and feedback. Notably, social interactions are usually not during the performance task itself. Regardless, the probability of emotional contagion with these frequent social interactions is critical for the subsequent team performance. This frequent and intense exchange of verbal and non-verbal information (e.g., instruction about the next play, emotions expressed by the body position) requires teammates to interpret these signals correctly and adjust their actions accordingly (Sebanz and Knoblich, 2009; Gweon and Saxe, 2013). For example, as mentioned above, player A might feel insecure after making a mistake. As a result, the teammates who have recognized player A’s emotions may try to overcompensate for their teammates. By noticing the latent support, player A may focus on improving the current game rather than brooding over a past mistake, ultimately regaining security.

Gaze behavior plays an essential role in transmitting emotions within social interactions because of its dual functionality (Gobel et al., 2015). This means that the eyes send information to another person and at the same time receive signals from the interacting person (Gobel et al., 2015).

The information sent by the eyes depends on the gaze’s direction (directed or averted) and duration and plays a crucial role in communication (Wirth et al., 2010; Canigueral and Hamilton, 2019). In detail, directed gaze refers to looking into somebody’s face. It signals that the sender desires an interaction and expresses positive emotions such as joy, anger, or affection (Foulsham et al., 2011). Instead, averted gaze involves, for example, looking at the floor while communicating with another person. It indicates the person’s unwillingness to communicate and represents negative emotions such as fear, worry, or shame (Argyle and Cook, 1976; Kleinke, 1986).

Previous research has shown that external stimuli such as stress or anxiety lead to gaze behavior changes as increased fixation on irrelevant stimuli and increased eye movement (Allsop and Gray, 2014; Vine et al., 2015). This is in line with the attentional control theory (ACT) (Corbetta and Shulman, 2002; Eysenck et al., 2007), which states that stress or anxiety is intended to reduce the relative influence of the top-down (goal-directed) system in favor of the bottom-up (stimulus-directed) system of attention. As a result, the individual’s attention is no longer outwardly focused on goal-related sources of information and a performance decline can be expected (e.g., Wilson et al., 2009; Noël and Kamp, 2012). In addition, Herten et al. (2017) examined participants’ gaze behavior in an interviewing situation and found shorter fixation durations on the face of interviewing committee members under a high-stress condition compared with a low-stress condition.

Despite the importance of social interactions in team sports and the crucial duality of the eye within this domain, studies dedicated to investigating social interaction in team sports context are scarce to date. Particularly, the examination of how gaze behavior is impacted by game performance pressure and whether inter-individual differences occur remains unclear in the literature. Indeed, so far most of the previous studies have focused on investigating the gaze behavior of individuals in their assigned tasks in a laboratory setting under varying stress conditions (e.g., Allsop and Gray, 2014; Allsop et al., 2016). A few studies have investigated the cooperative gaze behavior of teams in the laboratory (e.g., Bahrami et al., 2010; Neider et al., 2010), and an even smaller number have investigated the cooperative gaze behavior of groups in sports settings during the game (e.g., Fasold et al., 2018, 2021; Klatt et al., 2021a). Nevertheless, based on the dual function of the eyes, it seems reasonable that gaze behavior influences emotional contagion and, therefore, affects team performance. Thus, this study aimed to investigate gaze behavior in social interactions of expert beach volleyball teams in game-like situations and how it is affected by game performance pressure. Based on the literature, the study evaluates the hypothesis whether high game performance pressure lead to fewer fixations and shorter fixation durations (c.f., Allsop and Gray, 2014). Furthermore, shorter fixation durations on the teammates’ faces are hypothesized (cf. Herten et al., 2017).

## Materials and methods

### Participants

A total of 18 participants (nine females and nine males) were included in this study. The following criteria were used to include the participants into the study: (1) active beach volleyball players competing at least at state level; (2) practicing for more than 6 h per week; (3) more than 1 year of competitive training; and (4) normal or corrected-to-normal vision. Participants included in the statistical analysis were four female (*M*_*age*_ = 16.00 years, *SD*_*age*_ = 0.00) and three male (*M*_*age*_ = 22.60 years; *SD*_*age*_ = 3.78) beach volleyball players. For the remaining 11 participants, fulfillment of at least one condition was insufficient for further data analysis (e.g., recorded frames per second were not 30 or the video file was corrupted). This was mainly caused due to the dynamic nature of beach volleyball. The athletes included in the statistical analyses were engaged in competitive training and games for at least 2 years preceding the experiment (*M* = 2.79 years, *SD* = 1.35). They trained on average for 12.84 h (*SD* = 4.58) per week. Two athletes were a part of the c-squad (regional level) at the time of data collection. All participants had previously participated in the German Championships in their respective age categories. More detailed information about the participants is listed in Supplementary Table 1. The study was in accordance with the principles outlined by the World Medical Association’s Declaration of Helsinki and the Office of Research Ethics at the German Sport University Cologne (ethics proposal number: 184/2020). All participants and, if necessary, their legal guardians gave written consent to participate in this study voluntarily.

### Design

The game performance pressure manipulation was conducted in two different beach volleyball game forms. The low game performance pressure condition consisted of a standard beach volleyball set of 21 points. In contrast, under the high game performance pressure condition, the participants played nine short sets starting at 17:17, reaching 21 points to win in a best-of-9 mode. This manipulation was based on the findings of Marcelino et al. (2011) and Ramos et al. (2020), who found that final set moments were considered as high-pressure moments. Due to the shorter set length and the associated greater importance of each error right at the beginning of the set under the high game performance pressure condition, this form of play implicitly increases the game performance pressure on the athletes.

### Materials

The participants’ gaze behavior was assessed using four binocular mobile eye tracking systems (Kassner et al., 2014). Each mobile bundle consisted of a mobile phone (Motorola Moto Z2/Z3 Play) and a Pupil Core Headset (Pupil Labs GmbH, Berlin, Germany). The gaze accuracy is stated as 0.60° and the gaze precision as 0.02° (Pupil Labs GmbH, Berlin, Germany). The eye tracking data were recorded using the pupil mobile app on the mobile phone and simultaneously streamed and recorded *via* a Wireless Router (AVM FRITZ!Box 7590 Router) to a notebook (Dell Latitude 3510, 16 GB RAM, Intel Core i7), using the pupil capture app running on Windows 10. The routers were connected to the laptop *via* a LAN cable. Two eye tracking systems were streamed *via* the same router onto a notebook. Thus, two routers and two laptops were used. The eye tracking video was recorded with 30 frames per second.

### Procedure

Before participating in the experiment, participants warmed up as they would do before a competition, followed by a familiarization task wearing the eye tracking device. The familiarization task consisted of eight rallies including four side-out situations for each team. In the side-out situation, the opposing team serves. The own team has to receive the serve and set the ball and then attack. After the familiarization task, the participants took part in the two game performance pressure conditions (low vs. high) in a counterbalanced order. Thus, half of the teams started with the low and the other half with the high game performance pressure condition. Between these conditions, participants took a 10-min break. Before each game performance pressure condition, a *Manual Marker Calibration* (Kassner et al., 2014) was conducted: The participants stood in the middle of the beach volleyball court, while one examiner held a Pupil Calibration Marker v0.4 in his hands and stood one meter away from them. The participants were told to follow the Pupil Calibration Marker v0.4 with their eyes and not to move their heads, while the examiner followed a pre-defined route with the marker. After seven points, the participants also conducted a short in-game calibration instead of changing sides. Therefore, the participants stood in front of a Pupil Calibration Marker v0.4 placed on the side of their court. Participants focused their gaze on the midpoint of the marker while moving their heads up and down as well as to the left and right. One week prior to the testing, the *Team Cohesion Questionnaire [Fragebogen zur Mannschaftskohäsion]^1^* (Lau et al., 2003) and the expertise questionnaire were sent to be filled out by the participants until the day of the testing. The Team Cohesion Questionnaire was used to control for the possible influence of different relationship levels between team members on the study’s results. This is necessary because emotions are more easily transferred between team members with a good relationship than between team members with a poor relationship (cf. Tamminen et al., 2016). The expertise questionnaire only consisted of questions to ensure that the participants fulfilled the inclusion criteria.

### Data extraction

Social interactions between the rallies were tagged in the eye tracking videos (startpoint and endpoint were marked in the video). The starting point of the social interaction began when the previous rally was completed (the ball touched the ground) and ended when the teammates moved away from each other again to take their positions for the next rally. Thereafter, manual frame-by-frame analysis was used to analyze the athletes’ gaze behavior within these social interactions. This method has been successfully used by various researchers in previous investigations (see Patla and Vickers, 2003; Klatt et al., 2021a). We defined fixation as a gaze on the same area of interest (AOI: *face, upper body, lower body, and environment*) for more than 100 ms (more than three following frames) irrespective of these AOI moving in space (see Causer et al., 2010; Panuk et al., 2017). The AOI face, upper body, and lower body were defined as the teammate’s corresponding body parts. The AOI environment covered all other possible fixation points.

### Data analysis

We conducted a repeated measures MANOVA using Pillai’s Trace with game performance pressure level (low, high) as within-subject factor, participants (1–7) as between-subject factor, and the number of fixations as well as fixation duration on the areas of interest (*face, upper body, lower body, environment*) as dependent variables. In case of any multivariate effects, subsequent univariate tests were conducted. Greenhouse–Geisser adjustment was used to correct for violations of sphericity (if necessary) (O’Brien and Kaiser, 1985). For all tests, an alpha level was set at 0.05 and for effect size estimation eta square was used. A small effect was assumed for η^2^ = 0.01, a medium effect for η^2^ = 0.06, and a large effect for η^2^ = 0.14 (Cohen, 1988). The calculation was done using SPSS (version 28).

## Results

The mixed MANOVA showed multivariate significant effects of game performance pressure level [*Pillai’s Trace* = 0.09, F(8,192) = 2.43, *p* = 0.016, η^2^ = 0.014], participants [*Pillai’s Trace* = 0.84, F(48,1182) = 4.02, *p* < 0.001, η*^2^* = 0.14] as well as the game performance pressure level * participants interaction [*Pillai’s Trace* = 0.44, F(48,1182) = 1.97, *p* < 0.001, η*^2^* = 0.07].

*Game performance pressure level:* Subsequent univariant analyses showed that the game performance pressure level had a significant effect on fixation duration on the face [F(1,199) = 7.02, *p* = 0.009, η*^2^* = 0.03], the upper body [F(1,199) = 6.06, *p* = 0.015, η*^2^* = 0.03], and the number of fixations on the face [F(1,199) = 10.36, *p* = 0.002, η*^2^* = 0.05]. Under the high game performance pressure condition, participants fixated longer on the face and upper body (all *ps* < 0.05). Furthermore, the participants looked more frequently on the face (*p* < 0.05). All other univariate analyses did not show significant differences between the game performance pressure levels. All the means and standard deviation are shown in Table 1.

**TABLE 1:** Means and standard deviation of the fixation durations and numbers of fixations on the AOI face, upper body, lower body, and environment in relation to the game performance pressure condition.

	*Fixation duration [ms]*				*Number of fixations*			
		
	*High game performance pressure*		*Low game performance pressure*		*High game performance pressure*		*Low game performance pressure*	
				
	*M*	*SD*	*M*	*SD*	*M*	*SD*	*M*	*SD*
**Face**	315.01	493.39	199.35	375.32	0.61	0.78	0.42	0.60
**Upper body**	447.25	447.21	383.82	443.51	0.82	0.71	0.76	0.73
**Lower body**	51.78	207.38	37.86	147.36	0.09	0.32	0.09	0.331
**Environment**	1978.32	2447.03	1583.82	2192.75	1.14	0.79	1.05	0.87

**FIGURE 1:**
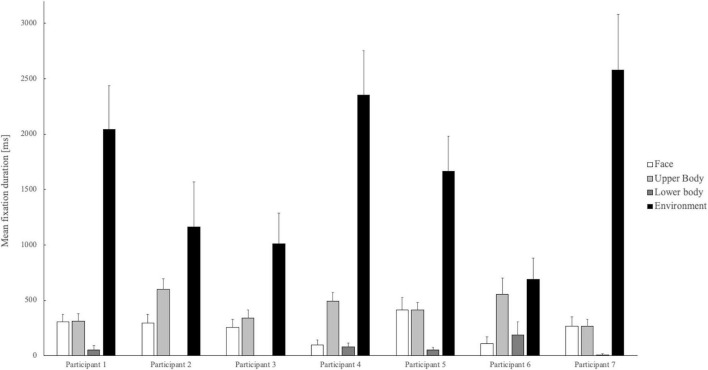
Mean fixation time of the players on the different areas of interest in ms. The error bars indicate the respective standard errors.

*Participants*: Univariate analyses showed that fixation duration on the team member’s face [F(6,199) = 4.27, *p* < 0.001, η*^2^* = 0.11], upper body [F(6,199) = 4.69, *p* < 0.001, η^2^ = 0.12], lower body [F(6,199) = 4.17, *p* < 0.001, η*^2^* = 0.11], and the environment [F(6,199) = 5.44, *p* < 0.001, η*^2^* = 0.14] differed statistically significant between the participants. The face fixation duration was significantly longer for participant 5 than for participant 4 (*p* < 0.001). For the upper body, participant 2 focused longer than participants 1, 3, and 7 (all *ps* < 0.05). Participant 6 fixated significantly longer on the teammate’s lower body in social interaction than participants 2, 3, and 7 (all *ps* < 0.005). Participants 2, 3, and 6 spent significantly less time on the environment than participants 4 and 7. For a graphical overview, the different fixation durations of the participants are shown in Figure 1. All the means and standard deviation are shown in Table 2.

**TABLE 2:** Means and standard deviation of the fixation durations of the participants on the AOI face, upper body, lower body, and environment included in the statistical analysis.

	*Fixation duration [ms]*							
	
	*Face*		*Upper body*		*Lower body*		*Environment*	
				
	*M*	*SD*	*M*	*SD*	*M*	*SD*	*M*	*SD*
**Participant 1**	304.76	410.04	311.43	397.37	54.76	238.23	2042.38	2347.99
**Participant 2**	292.97	433.08	601.15	499.80	0.00	0.00	1164.94	2180.75
**Participant 3**	258.05	375.50	342.53	381.29	0.00	0.00	1009.77	1496.12
**Participant 4**	97.97	297.89	495.12	504.30	81.71	212.44	2356.10	2566.96
**Participant 5**	416.15	622.65	413.54	368.84	51.04	151.10	1665.63	1803.19
**Participant 6**	110.61	202.91	554.55	487.59	189.39	388.48	692.42	627.244
**Participant 7**	268.39	440.35	265.52	342.04	8.62	65.65	2580.46	2703.26

**FIGURE 2:**
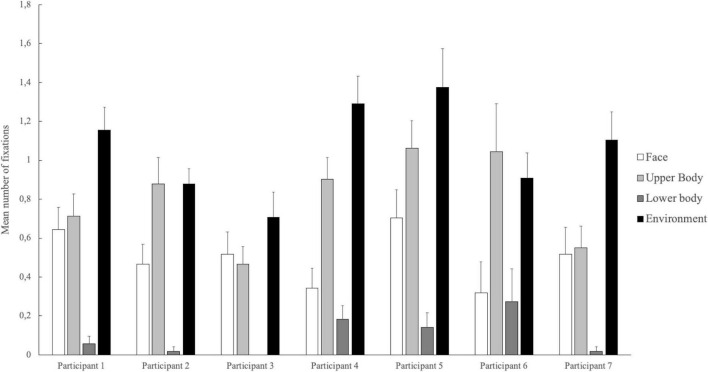
Mean fixation count on the different areas of interest for each player. The error bars indicate the respective standard errors.

For the number of fixations, the univariate tests revealed significant differences between participants’ fixations on the face [F(6,199) = 2.59, *p* = 0.019, η*^2^* = 0.07], on the upper body [F(6,199) = 6.77, *p* < 0.001, η*^2^* = 0.17], on the lower body [F(6,199) = 4.15, *p* < 0.001, η*^2^* = 0.11], and on the environment [F(6,199) = 5.67, *p* = 0.001, η*^2^* = 0.15]. Participant 4 fixated on the teammate’s face significantly more often than participant 5 (*p* = 0.028). On the teammate’s upper body, participant 3 showed significantly fewer fixations than participants 2, 4, 5, and 6 (all *ps* < 0.05). Also, in contrast to participant 7, participants 4 and 5 focused on the upper body significantly more often. Participants 3 fixated on the lower body in social interaction significantly less often than participants 4 and 6 (all *ps* < 0.05). For the number of fixations on the environment, there was a significantly lower number for participants 2 and 3 compared with participants 4 and 5 (all *ps* < 0.05). In addition, participant 3 had lower fixation numbers than participant 1 (*p* < 0.001) (see Figure 2). All the means and standard deviation are shown in Table 3.

**TABLE 3:** Means and standard deviation of the number of fixations of the participants on the AOIs face, upper body, lower body, and environment included in the statistical analysis.

	*Number of fixations*							
	
	*Face*		*Upper body*		*Lower body*		*Environment*	
		
	*M*	*SD*	*M*	*SD*	*M*	*SD*	*M*	*SD*
**Participant 1**	0.64	0.69	0.71	0.67	0.06	0.23	1.16	0.69
**Participant 2**	0.47	0.56	0.88	0.73	0.02	0.13	0.88	0.42
**Participant 3**	0.52	0.61	0.47	0.48	0	0	0.71	0.70
**Participant 4**	0.34	0.66	0.90	0.72	0.18	0.45	1.29	0.90
**Participant 5**	0.70	0.82	1.06	0.80	0.14	0.43	1.38	1.14
**Participant 6**	0.32	0.53	1.05	0.82	0.27	0.56	0.91	0.43
**Participant 7**	0.52	0.75	0.55	0.60	0.02	0.13	1.10	0.79

**FIGURE 3:**
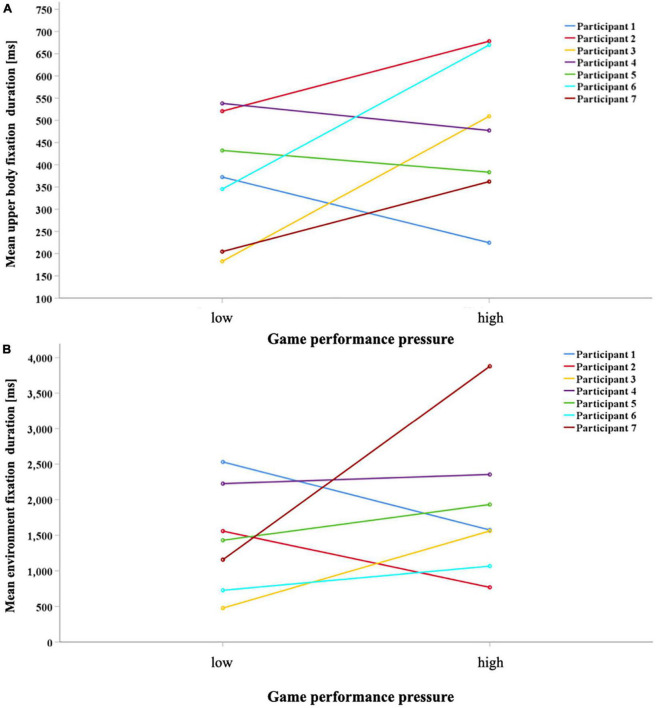
Each participant’s mean fixation duration depending on the stress level on the upper body **(A)** and the environment **(B)**.

Game performance pressure level * Participants interaction: The univariate analyses of the interaction between the participants and game performance pressure level showed that the level of game performance pressure affected participants’ gaze differently with regard to upper body fixation durations [F(6,199) = 2.86, *p* = 0.011, η*^2^* = 0.08] and fixation durations of the environment [F(6,199) = 5.00, *p* < 0.001, η*^2^* = 0.13]. Participants 1, 4, and 5 spent less time focusing on the upper body under the high game performance pressure condition. In contrast, all others looked longer at their partner’s upper body under the high game performance pressure condition. Considering the fixation durations, only participants 1 and 2 focused for a shorter duration due to increased game performance pressure. These individual differences are shown in Figure 3. All other univariate analyses did not show significant differences between the game performance pressure levels (all *ps* > 0.05).

**FIGURE 4:**
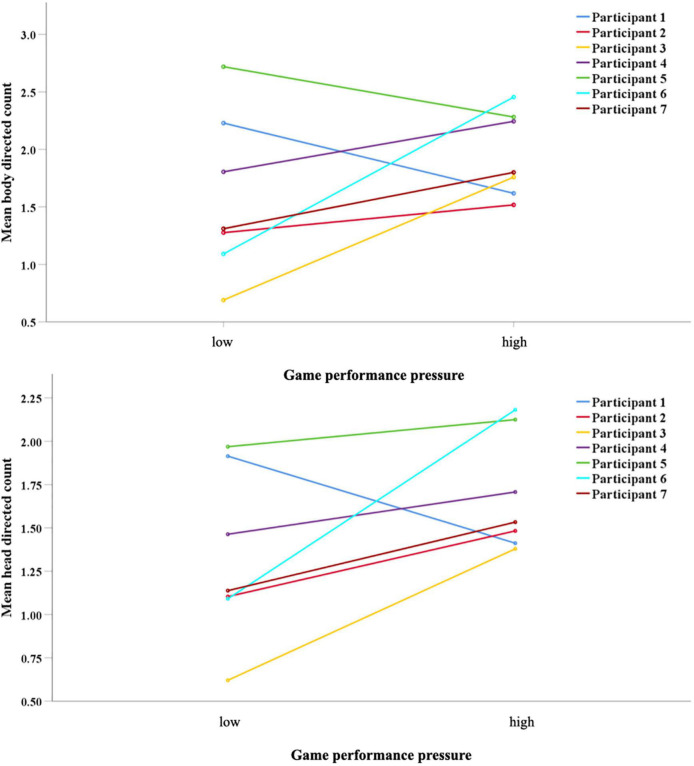
Mean fixation numbers on the face **(A)** and the environment **(B)** per participant for the high- and low game performance pressure condition.

For the number of fixations, game performance pressure level * participant interaction indicated significant differences for the AOI face [F(6,199) = 2.70, *p* = 0.015, η*^2^* = 0.07] and environment [F(6,199) = 2.73, *p* = 0.014, η*^2^* = 0.08]. All but participant 1 fixated more often on the face under the high game performance pressure condition. Considering the AOI environment, only participants 1 and 5 showed a reduced frequency under the high game performance pressure condition. While this is the case, a reduction in the variance of fixation count can also be observed (see Figure 4).

*Results Team Cohesion Questionnaire:* The task cohesion (*M* = 5.84, *SD* = 0.38) and social cohesion (*M* = 5.37, *SD* = 0.80) values were high with slight variances. Despite the homogeneously high values, the female participants showed descriptively slightly higher values than the male participants (see Supplementary Table 2).

## Discussion

This exploratory study aimed to examine whether a higher game performance pressure level leads to increased eye movements and shorter fixation durations in the social interaction between players between the rallies. The results showed that participants fixate significantly longer on the upper body and the face under the high game performance pressure conditions. Further, the participants looked more frequent at their teammates’ faces. No other changes in gaze behavior were found due to game performance pressure level manipulation. The analysis also showed that there are individual differences between the participants.

Concerning the longer and more frequent fixation on the teammates’ faces and longer duration on the teammates’ upper body under the high game performance pressure condition, it seems that especially the face takes on a special role within the social interaction between the rallies. On the one hand, faces send a lot of emotional stimuli about the other person (Bahrick and Lickliter, 2014; Caulfield et al., 2016; Crivelli et al., 2016). Recognizing the emotions of the teammate is important to possibly provide support if the teammate needs it. However, it can also lead to emotion contagion, which may lead to a Collective Team Collapse, if negative emotions are transferred (Wergin et al., 2018). On the other hand, the visual system also plays a crucial role in the reception of auditory information (Klatt and Smeeton, 2020). For example, directing the gaze to the speaker improves the auditory stimulus reception (Dodd, 1977; Summerfield, 1987). Especially in tight game situations, understanding the verbal information of the teammate correctly seems to be decisive, as any loss of points can lead to defeat. Interestingly, individual differences in gaze behavior and success rate seem to indicate a pattern underlining this assumption. The players winning most of the rallies in this study (participants 5 and 6) increased the duration and number of fixations on the face under the high game performance pressure condition. In contrast, the player losing the most rallies in this study (participant 1) fixated the teammate’s face less and for a shorter time. This could lead to the assumption that the pattern of participants 5 and 6 increases the quality of social interaction. This increased quality could be needed to generate better emotional and game-related feedback. In total, the better receiving of auditory information and teammate’s emotional state suggest that the adaptation of gaze behavior has a functional role. However, these results contradict the previous research findings, which found that individuals tend to adopt an averted gaze behavior (less fixation on the face) in stressful interview situations (Herten et al., 2017). In this situation, faces may be perceived as aversive stimuli. Therefore, the gaze was shifted from the threatening input (Mogg and Bradley, 1998; Mogg et al., 2000; Wilson and MacLeod, 2003) and direct to different objects in the environment rather than the face of the pressure-inducing person (Herten et al., 2017). In beach volleyball teams, the partner should not be considered as an aversive stimulus. Instead, the teammates had to work as a team toward a common goal, which differs from the interview situations investigated by Herten et al. (2017). Supplementary Table 2 also emphasizes that the teammates are not considered as an aversive stimulus by showing that all teams had positive relationships. These findings indicate that compared with a situation where the other person is regarded as an aversive stimulus and face fixations are reduced, in a teamwork scenario, participants increase their duration and frequency of fixations on the partner’s face to try and achieve better results.

According to the inter-individual difference, the results indicate that gaze behavior in social interaction is also affected by factors such as personality traits and socializing. Hence, in this study players showed inter-individual strategies to cope with the high game performance pressure condition regarding their gaze behavior. Notably, participant 5 had a distinct pattern of change in gaze behavior due to increased game performance pressure compared with other players. Participant 5 had a reduction in the total number of fixations, but an increased number and duration of fixations on the face. These results suggest that participant 5 became more focused on her teammate, allowing for emotional feedback and communication to occur more often and for longer than under the low-stress condition. In contrast to this strategy, participant 1 had decreased face fixation numbers and duration under the high game performance pressure condition. The changes in gaze behavior of participant 1 may have been the result of poor performance, which is emphasized by the lowest percentage of subsequent rallies won (34%) under the high game performance pressure condition. Due to this poor performance, it is possible that participant 1 averted his gaze caused by his own emotions such as fear or worry of making the next mistake (see Adams and Kleck, 2005). The teammate (participant 3) showed no significant changes in gaze behavior, assuming that these emotions were not transferred. So far, most studies have focused on mean group values, but not on individual differences (e.g., Herten et al., 2017; Timmis et al., 2018). However, it must be mentioned that the participants measured in this exploratory study were young competitive athletes, but not elite athletes. It could be that elite athletes have a higher experience with game performance pressure and may adapt their gaze differently. Future research should therefore focus on the analysis of individual differences in gaze behavior in social interactions and in sport with a specific focus on elite athletes (see Dicks et al., 2016).

## Conclusion

The study suggests a common change in gaze behavior in beach volleyball teams due to increased game performance pressure. An increased number and duration of fixations on the partner’s face were found, possibly seeking emotional and game-related feedback, indicating the need for more frequent and prolonged interactions. Furthermore, longer fixation duration and higher numbers of fixations on the face could also have a functional role in the communication between the teammates.

In practice, coaches may want to encourage players to increase the quantity and quality (directed head and fixations on the face) of social interactions between the rallies. Improved social interaction could lead to earlier recognition of negative emotions of the teammate, and counteracting this can make the occurrence of a collective team collapse less likely. Routines can increase the quantity of gaze on the face of the teammate between the rallies, such as the athletes high-fiving each other and then having to look at each other’s faces regardless of how the last rally went. Another way could be to inform the players about how important it is to gather the information sent by the teammate’s face. Furthermore, verbal and non-verbal communication seems to be a crucial aspect to consider when forming a beach volleyball team.

It needs to be mentioned that the results of the current study are mainly exploratory and only traced back to a small sample size, which restricts the generalization of these findings. Moreover, it can be assumed that manipulating the game performance pressure level in this controlled setting is not comparable to game performance pressure during real competitions. It is possible that the players communicate differently in this experimental setting than they would in a game with spectators. Therefore, further research is needed to understand how gaze behavior changes due to stressful situations in social interactions in natural sport settings. It also seems to make sense to focus on phases of the game where social interaction is possible for a longer period, like time-outs, rather than only between the rallies. Nevertheless, this study opens the door to a new research field and raises new research topics within this area.

## Data availability statement

The raw data supporting the conclusions of this article will be made available by the authors, without undue reservation.

## Ethics statement

The studies involving human participants were reviewed and approved by Office of Research Ethics German Sport University Cologne. Written informed consent to participate in this study was provided by the participants’ legal guardian/next of kin.

## Author contributions

L-MR, JK, MB, BN, and SK conceptualized the project. AN wrote the first draft of the manuscript and conducted the data recordings. MV analyzed the data. SK, MB, and BN revised all drafts and wrote parts of the manuscript. All authors contributed to the article and approved the submitted version.

## Footnotes

1 For reliability analysis, Cronbach’s alpha was calculated and showed satisfying results of the social cohesion (0.84) and the task cohesion (0.81) (Lau et al., 2003). The average retest reliability coefficient for these subscales is rtt = 0.73 and the average intercorrelation is *r* = 0.54 (Lau, 2005).

## Supplementary material

Supplementary Figure 1 Mean numbers of fixations for each AOI. Significant Wilcoxon’s test results (*p* < 0.013) comparing the low- and high-stress conditions. Error bars represent the standard errors of the means.

Supplementary Figure 2 Mean total number of fixations for each AOI. Significant Wilcoxon’s test results (*p* < 0.01) comparing the low- and high-stress conditions. Error bars represent the standard errors of the means.

## References

[B1] Adams R. B. Jr. Kleck R. E. (2005). Effects of direct and averted gaze on the perception of facially communicated emotion. *Emotion* 5 3–11. 10.1037/1528-3542.5.1.3 15755215

[B2] Allsop J. Gray R. (2014). Flying under pressusre: Effects of anxiety on attention and gaze behavior in aviation. *J. Appl. Res. Memory Cogn.* 3 63–71. 10.1016/j.jarmac.2014.04.010

[B3] Allsop J. Gray R. Bulthoff H. H. And Chuang L. (2016). Effects of anxiety and cognitive load on instrument scanning behavior in a flight simulation. *IEEE Second Workshop Eye Track. Visualiz.* 2016 55–59. 10.1109/ETVIS.2016.7851167

[B4] Argyle M. Cook M. (1976). *Gaze and Mutual Gaze.* Cambridge: Cambridge University Press.

[B5] Bahrami B. Olsen K. Latham P. E. Roepstorff A. Rees G. Frith C. D. (2010). Optimally interacting minds. *Science* 329 1081–1085. 10.1126/science.1185718 20798320 PMC3371582

[B6] Bahrick L. E. Lickliter R. (2014). Learning to attend selectively: The dual role of intersensory redundancy. *Current Directions In Psychological Science* 23 414–420. 10.1177/0963721414549187 25663754 PMC4316375

[B7] Barsade S. G. Gibson D. E. (2012). Group affect: Its influence on individual and group outcomes. *Curr. Direct. Psychol. Sci.* 21 119–123. 10.1177/0963721412438352

[B8] Boss M. Kleinert J. (2015). Explaining social contagion in sport applying Heider’s balance theory: first experimental results. *Psychol. Sport Exerc*. 16, 160–169. 10.1016/j.psychsport.2014.10.006

[B9] Canigueral R. Hamilton A. F. C. (2019). The role of eye gaze during natural social interactions in typical and autistic people. *Front. Psychol.* 10:560. 10.3389/fpsyg.2019.00560 30930822 PMC6428744

[B10] Caulfield F. Ewing L. Bank S. Rhodes G. (2016). Judging trustworthiness from faces: Emotion cues modulate trustworthiness judgments in young children. *Br. J. Psychol.* 107 503–518. 10.1111/bjop.12156 26493772

[B11] Causer J. Bennett S. J. Holmes P. S. Janelle C. M. Williams A. M. (2010). Quiet eye duration and gun motion in elite shotgun shooting. *Med. Sci. Sports Exerc.* 42 1599–1608. 10.1249/MSS.0b013e3181d1b059 20139787

[B12] Clark H. H. Krych M. A. (2004). Speaking while monitoring addressees for understanding. *J. Memory Lang.* 50 62–81. 10.1016/j.jml.2003.08.004

[B13] Cohen J. (1988). “Chapter 8. the analysis of variance and covariance,” in *Statistical Power Analysis For The Behavioral Sciences*, (New York: Routledge Academic), 273–406. 10.1016/B978-0-12-179060-8.50013-X

[B14] Corbetta M. Shulman G. L. (2002). Control of goal-directed and stimulus-driven attention in the brain. *Nat. Rev. Neurosci.* 3 201–215. 10.1038/nrn755 11994752

[B15] Crivelli C. Jarillo S. Russell J. A. Fernández-Dols J.-M. (2016). Reading emotions from faces in two indigenous societies. *J. Exp. Psychol. General* 145 830–843. 10.1037/xge0000172 27100308

[B16] Dicks M. Button C. Davids K. Chow J. Y. Der Kamp J. (2016). Keeping an eye on noisy movements: On different approaches to perceptual-motor skill research and training. *Sports Med.* 47 575–581. 10.1007/s40279-016-0600-3 27497599

[B17] Dodd B. (1977). The role of vision in the perception of speech. *Perception* 6 31–40. 10.1068/p060031 840618

[B18] Eysenck M. W. Derakshan N. Santos R. Calvo M. G. (2007). Anxiety and cognitive performance: Attentional control theory. *Emotion* 7:336. 10.1037/1528-3542.7.2.336 17516812

[B19] Fasold F. Nicklas A. Seifriz F. Schul K. Noël B. Aschendorf P. (2021). Gaze coordination of groups in dynamic events–a tool to facilitate analyses of simultaneous gazes within a team. *Front. Psychol.* 12:816. 10.3389/fpsyg.2021.656388 33815236 PMC8009972

[B20] Fasold F. Noël B. Wolf F. Hüttermann S. (2018). Coordinated gaze behaviour of handball referees: A practical exploration with focus on the methodical implementation. *Mov. Sport Sci. Sci. Motric.* 2018 71–79. 10.1051/sm/2018029

[B21] Foulsham T. Walker E. Kingstone A. (2011). The where, what and when of gaze allocation in the lab and the natural environment. *Vision Res.* 51 1920–1931. 10.1016/j.visres.2011.07.002 21784095

[B22] Gobel M. S. Kim H. S. Richardson D. C. (2015). The dual function of social gaze. *Cognition* 136 359–364. 10.1016/j.cognition.2014.11.040 25540833

[B23] Gweon H. Saxe R. (2013). “Developmental cognitive neuroscience of theory of mind,” in *Neural circuit development and function in the brain*, Vol. 3, eds Rubenstein J. L. R. Rakic P. (Amsterdam: Elsevier), 367–377. 10.1016/B978-0-12-397267-5.00057-1

[B24] Hatfield E. Bensman L. Thornton P. D. Rapson R. L. (2014). New perspectives on emotional contagion: A review of classic and recent research on facial mimicry and contagion. *Int. Int. J. Personal Relations.* 8 159–179. 10.5964/ijpr.v8i2.162 33680184

[B25] Herrando C. Constantinides E. (2021). Emotional contagion: A brief overview and future directions. *Front. Psychol.* 2021:12. 10.3389/fpsyg.2021.712606 34335425 PMC8322226

[B26] Herten N. Otto T. Wolf O. T. (2017). The role of eye fixation in memory enhancement under stress - an eye tracking study. *Neurobiol. Learn. Mem.* 140 134–144. 10.1016/j.nlm.2017.02.016 28267591

[B27] Hill D. M. Shaw G. (2013). A qualitative examination of choking under pressure in team sport. *Psychol. Sport Exerc.* 14 103–110. 10.1016/j.psychsport.2012.07.008

[B28] Jordan J. S. Knoblich G. (2004). Spatial perception and control. *Psychon. Bull. Rev.* 11 54–59. 10.3758/BF03206460 15116986

[B29] Kassner M. Patera W. Bulling A. (2014). “Pupil: An open source platform for pervasive eye tracking and mobile gaze-based interaction,” in *Proceedings Of The 2014 Acm International Joint Conference On Pervasive And Ubiquitous Computing: Adjunct Publication*, 1151–1160. 10.1145/2638728.2641695

[B30] Klatt S. Noël B. Nicklas A. Schul K. Seifriz F. Schwarting A. (2021a). Gaze behavior and positioning of referee teams during three-point shots in basketball. *Appl. Sci.* 11:6648. 10.3390/app11146648

[B31] Klatt S. Noël B. Schwarting A. Heckmann L. Fasold F. (2021b). Adaptive gaze behavior and decision making of penalty corner strikers in field hockey. *Front. Psychol.* 12:674513. 10.3389/fpsyg.2021.674511 PMC8366230 34408695

[B32] Klatt S. Smeeton N. J. (2020). Visual auditory information during decision making in sport. *J. Sport Exerc. Psychol.* 42 15–25. 10.1123/jsep.2019-0107 31883505

[B33] Kleinke C. L. (1986). Gaze eye contact: A research review. *Psychol. Bull.* 100:78. 10.1037/0033-2909.100.1.78 3526377

[B34] Knoblich G. Jordan J. S. (2002). The mirror system joint action. *Adv. Consciou. Res.* 42 115–124. 10.1075/aicr.42.10kno 33486653

[B35] Knoblich G. Sebanz N. (2008). Evolving intentions for social interaction: From entrainment to joint action. *Philos. Trans. R. Soc. Biol. Sci.* 363 2021–2031. 10.1098/rstb.2008.0006 18292061 PMC2606699

[B36] Lau A. Stoll O. Hoffmann A. (2003). Diagnostik und stabilität der mannschaftskohäsion in den sportspielen. *Leipziger Sportwissenschaftliche Beiträge* 44 1–24.

[B37] Lau A. (2005). Das teamentwicklungstraining – ein systemisches konzept für die mannschaftssportspiele. *Leipziger Sportwissenschaftliche Beiträge* 46 64–82.

[B38] Marcelino R. Mesquita I. Sampaio J. (2011). Effects of quality of opposition match status on technical tactical performances in elite volleyball. *J. Sports Sci.* 29 733–741. 10.1080/02640414.2011.552516 21424980

[B39] Marsh K. L. Richardson M. J. Baron R. M. Schmidt R. (2006). Contrasting approaches to perceiving acting with others. *Ecol. Psychol.* 18 1–38. 10.1207/s15326969eco1801_1

[B40] Mogg K. Bradley B. P. (1998). A cognitive-motivational analysis of anxiety. *Behav. Res. Ther.* 36 809–848. 10.1016/S0005-7967(98)00063-1 9701859

[B41] Mogg K. Mcnamara J. Powys M. Rawlinson H. Seiffer A. Bradley B. P. (2000). Selective attention to threat: A test of two cognitive models of anxiety. *Cogn. Emot.* 14 375–399. 10.1080/026999300378888

[B42] Moll T. Jordet G. Pepping G.-J. (2010). Emotional contagion in soccer penalty shootouts: Celebration of individual success is associated with ultimate team success. *J. Sports Sci.* 28 983–992. 10.1080/02640414.2010.484068 20544488

[B43] Neider M. B. Chen X. Dickinson C. A. Brennan S. E. Zelinsky G. J. (2010). Coordinating spatial referencing using shared gaze. *Psychon. Bull. Rev.* 17 718–724. 10.3758/PBR.17.5.718 21037172

[B44] Noël B. Kamp J. V. D. (2012). Gaze behaviour during the soccer penalty kick: An investigation of the effects of strategy anxiety. *Int. J. Sport Psychol.* 43 326–345.

[B45] O’Brien R. G. Kaiser M. K. (1985). Manova method for analyzing repeated measures designs: An extensive primer. *Psychol. Bull.* 97:316. 10.1037/0033-2909.97.2.316 3983301

[B46] Panuk D. Vickers J. N. Hopkins W. G. (2017). Quiet eye predicts goaltender success in deflected ice hockey shots. *Eur. J. Sport Sci.* 17 93–99. 10.1080/17461391.2016.1156160 26949176

[B47] Patla A. E. Vickers J. N. (2003). How far ahead do we look when required to step on specific locations in the travel path during locomotion? *Exp. Brain Res.* 148 133–138. 10.1007/s00221-002-1246-y 12478404

[B48] Ramos A. Coutinho P. Ribeiro J. Fernandes O. Davids K. Mesquita I. (2020). Increasing tactical complexity to enhance the synchronization of collective behaviours: An action-research study throughout a competitive volleyball season. *J. Sports Sci.* 38 2611–2619. 10.1080/02640414.2020.1794265 32691698

[B49] Sebanz N. Bekkering H. Knoblich G. (2006). Joint action: Bodies minds moving together. *Trends Cogn. Sci.* 10 70–76. 10.1016/j.tics.2005.12.009 16406326

[B50] Sebanz N. Knoblich G. (2009). Prediction in joint action: What, when, and where. *Top. Cogn. Sci.* 1, 353–367. 10.1111/j.1756-8765.2009.01024.x 25164938

[B51] Summerfield Q. (1987). “Some preliminaries to a comprehensive account of audio-visual speech perception,” in *The Psychology Of Lip Reading*, ed. Eye H. (Hillsdale, NJ: Lawrence Erlbaum Associates), 3–51.

[B52] Tamminen K. A. Palmateer T. M. Denton M. Sabiston C. Crocker P. R. E. Eys M. (2016). Exploring emotions as social phenomena among canadian varsity athletes. *Psychol. Sport Exerc.* 27 28–38. 10.1016/j.psychsport.2016.07.010

[B53] Timmis M. A. Piras A. Van Paridon K. N. (2018). keep your eye on the ball: The impact of an anticipatory fixation during successful unsuccessful soccer penalty kicks. *Front. Psychol.* 9:2058. 10.3389/fpsyg.2018.02058 30429808 PMC6220034

[B54] Vine S. J. Uiga L. Lavric A. Moore L. J. Tsaneva-Atanasova K. Wilson M. R. (2015). Individual reactions to stress predict performance during a critical aviation incident. *Anxiety Stress Coping Int. J.* 28 467–477. 10.1080/10615806.2014.986722 25396282

[B55] Wergin V. V. Zimanyi Z. Mesagno C. Beckmann J. (2018). When suddenly nothing works anymore within a team - causes of collective sport team collapse. *Front. Psychol.* 9:2115. 10.3389/fpsyg.2018.02115 30459685 PMC6232390

[B56] Wilson E. MacLeod C. (2003). Contrasting two accounts of anxiety-linked attentional bias: Selective attention to varying levels of stimulus threat intensity. *J. Abnormal Psychol.* 112:212. 10.1037/0021-843X.112.2.212 12784830

[B57] Wilson M. R. Wood G. Vine S. J. (2009). Anxiety, attentional control, performance impairment in penalty kicks. *J. Sport Exerc. Psychol.* 31 761–775. 10.1123/jsep.31.6.761 20384011

[B58] Wirth J. H. Sacco D. F. Hugenberg K. Williams K. D. (2010). Eye gaze as relational evaluation: Averted eye gaze leads to feelings of ostracism relational devaluation. *Personali. Soc. Psychol. Bull.* 36 869–882. 10.1177/0146167210370032 20505162

[B59] Wolf D. Mittelberg I. Rekittke L.-M. Bhavsar S. Zvyagintsev M. Haeck A. (2018). Interpretation of social interactions: Functional imaging of cognitive-semiotic categories during naturalistic viewing. *Front. Hum. Neurosci.* 2018:12. 10.3389/fnhum.2018.00296 30154703 PMC6102316

