# Supplementary figures and images for: Gaze behavior in social interactions between beach volleyball players—An exploratory approach
Source: Front Psychol. 2022 Oct 11;13:945389. doi: 10.3389/fpsyg.2022.945389 (PMC9592831; doi:10.3389/fpsyg.2022.945389)

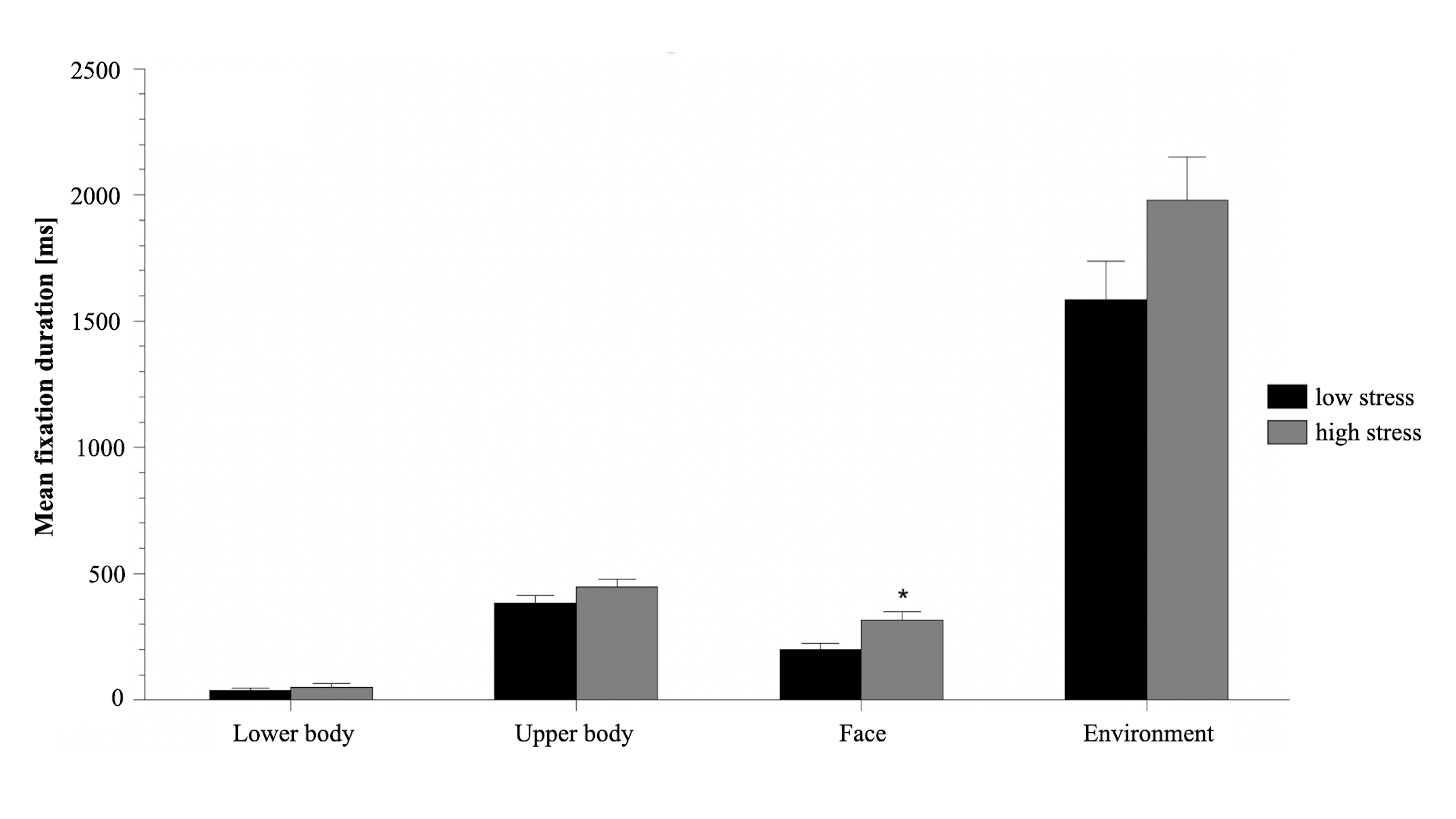

Supplement: Supplementary Figure 1 — Mean numbers of fixations for each AOI. Significant Wilcoxon’s test results (p < 0.013) comparing the low- and high-stress conditions. Error bars represent the standard errors of the means. [file Image_1.TIFF]

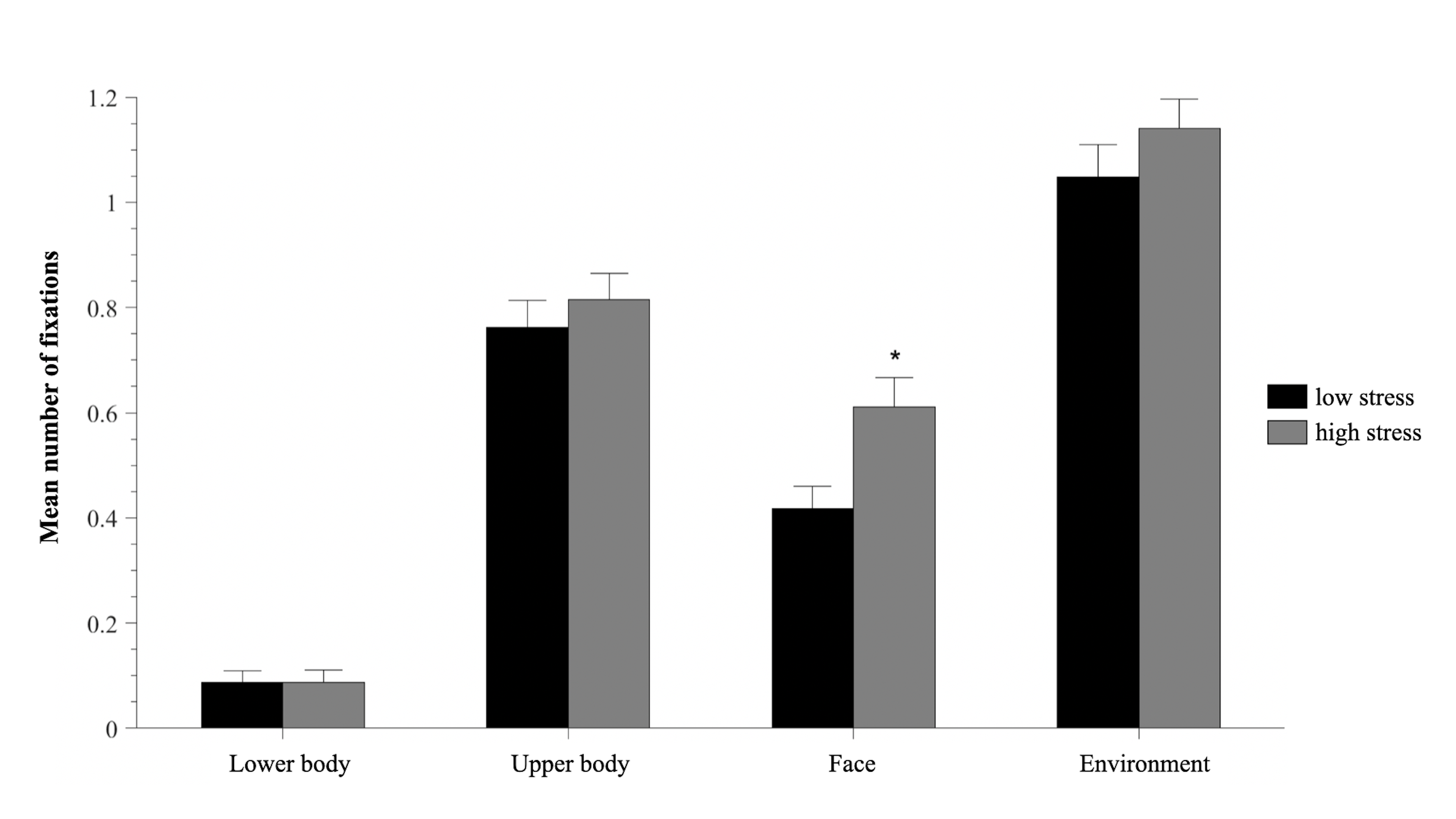

Supplement: Supplementary Figure 2 — Mean total number of fixations for each AOI. Significant Wilcoxon’s test results (p < 0.01) comparing the low- and high-stress conditions. Error bars represent the standard errors of the means. [file Image_2.TIFF]
