# Supplementary material for: Gaze behavior in social interactions between beach volleyball players—An exploratory approach
Source: Front Psychol. 2022 Oct 11;13:945389. doi: 10.3389/fpsyg.2022.945389 (PMC9592831; doi:10.3389/fpsyg.2022.945389)
Supplement: Supplementary file 2 [file Data_Sheet_1.docx]

Table S1: Expertise information of participants included into the analysis.

	Age	*Gender*	*Training/week [min]*	*Competitive Training [years]*	*Competing at national level*	*Competing at regional level*
**Participant 1**	*16*	*m*	*660*	*2*	*no*	*yes*
**Participant 2**	18	m	540	4	yes	yes
**Participant 3**	16	f	420	2	no	no
**Participant 4**	16	f	420	3	no	no
**Participant 5**	16	f	480	2.5	yes	yes
**Participant 6**	16	f	480	1	no	no
**Participant 7**	23	m	720	5	yes	yes

Table S2: Results of the Team Cohesion Questionnaire.

	Total sample *(N = 18, f =9, m = 9)*		***Sample included into inferential statistic*** *(N = 7; f = 4, m = 3)*	
	M	*SD*	*M*	*SD*
**Task cohesion**				
all	5.64	0.68	5.84	0.38
female	5.83	0.37	5.70	0.42
male	5.44	0.87	6.03	0.25
**Social cohesion**				
all	5.35	0.82	5.37	0.80
female	5.59	0.74	5.75	0.43
male	5.11	0.86	4.85	0.97
